# Efficacy and safety of anti-CD20 monoclonal antibody therapy for autoimmune nodopathies: a systematic review and meta-analysis

**DOI:** 10.3389/fneur.2026.1759210

**Published:** 2026-02-23

**Authors:** Zijie Tao, Yuhang Jiang, Qiyi Gui, Jie Ma

**Affiliations:** 1 Jiangsu Key Laboratory of Laboratory Medicine, Department of Immunology and Laboratory Medicine, School of Medicine, Jiangsu University, Zhenjiang, Jiangsu, China; 2 The First Clinical Medical College, Hubei University of Medicine, Shiyan, Hubei, China

**Keywords:** anti-CD20 monoclonal antibody, autoimmune nodopathy, chronic inflammatory demyelinating polyradiculoneuropathy, contactin–1, meta-analysis, neurofascin-155, ofatumumab, rituximab

## Abstract

**Background:**

Autoimmune nodopathy (AN) is a distinct CIDP-like entity defined by its poor response to standard treatments, including IVIG. The efficacy and safety of anti-CD20 monoclonal antibodies, a potential mechanism-based therapy, have not been quantitatively synthesized.

**Objective:**

To systematically evaluate and quantitatively synthesize the efficacy and safety of anti-CD20 monoclonal antibody therapy in patients with AN.

**Methods:**

A comprehensive literature search was conducted across PubMed, Web of Science, Cochrane Library, Embase, and ClinicalTrials.gov from inception to August 4, 2025. Studies reporting clinical outcomes of AN patients treated with anti-CD20 agents were included. A generalized linear mixed model (GLMM) was employed to estimate pooled response rates.

**Results:**

Twenty-nine studies comprising 118 patients were included. In the descriptive synthesis, most reports described physician-assessed clinical improvement after anti-CD20 therapy. For quantitative pooling, we restricted the meta-analysis to studies reporting standardized, objective scale-based outcomes (*n =* 100), yielding a pooled clinical response rate of 92.0% (95% CI, 84.8–95.9%, I^2^ = 0%). Subgroup analyses demonstrated sustained responsiveness in patients with anti-NF155 (95.2%) and anti-CNTN1 (88.9%) autoantibodies. Adverse events were recorded in 8.5% of patients (10/118), primarily consisting of mild infusion-related reactions. However, two fatalities (1.7%) associated with severe infection or comorbidities were noted.

**Conclusion:**

Anti-CD20 therapy has shown high efficacy in treating AN that is refractory to conventional treatments. However, due to the observational nature of the available data and the lack of randomized controlled trials, these results should be interpreted with caution and are not yet practice-changing. Further prospective, controlled studies are needed to better define the treatment’s efficacy, optimal dosing strategies, and long-term safety.

## 1 Introduction

Chronic inflammatory demyelinating polyradiculoneuropathy (CIDP) is a heterogeneous autoimmune neuropathy (1). Traditionally, patients are treated with corticosteroids and intravenous immunoglobulin (IVIG) as first-line therapies (2). However, a significant number of patients remain refractory to these conventional treatments, suggesting a different underlying pathophysiology (3). Recently, the 2021 European Academy of Neurology/Peripheral Nerve Society (EAN/PNS) guidelines formally introduced “autoimmune nodopathy” (AN) as a new diagnostic category, distinct from classic CIDP (2).

This distinct entity, AN, is defined by pathogenic antibodies targeting both nodal proteins (such as neurofascin-140/186) and paranodal proteins, including neurofascin-155 (NF155), contactin-1 (CNTN1), and contactin-associated protein 1 (CASPR1) (3–6). Pathologically, AN differs significantly from classic CIDP. In classic CIDP, nerve injury is caused by macrophage-mediated demyelination. In contrast, AN is frequently associated with IgG4 autoantibodies. According to recent reviews, IgG4 antibodies are functionally monovalent and do not activate the complement system or inflammatory cells. Instead, they directly block protein–protein interactions at the node of Ranvier (6–10). This non-inflammatory mechanism explains why conventional immunomodulatory therapies like IVIG and corticosteroids are often ineffective or only transiently effective in patients with AN (11, 12). Therefore, B-cell depleting therapies, such as rituximab, which reduce the production of pathogenic antibodies, have emerged as a more logical therapeutic strategy.

Although rituximab is increasingly used for these AN patients, current evidence mainly comes from case reports and small cohort studies (13–17). A previous meta-analysis reported a 75% response rate for rituximab in general CIDP patients. However, it did not specifically analyze the efficacy and safety of anti-CD20 therapies in the newly defined AN population (18). Therefore, we performed this systematic review and single-arm meta-analysis to quantitatively evaluate the efficacy and safety of anti-CD20 monoclonal antibodies in patients with AN, with a focus on anti-NF155 and anti-CNTN1 subgroups.

## 2 Methods

This systematic review and meta-analysis was performed according to the methodology recommended by the PRISMA statement (19).

### 2.1 Search strategy

A comprehensive literature search was conducted across major electronic databases, including PubMed, Web of Science, Cochrane, Embase and ClinicalTrials.gov, from their inception to August 4, 2025. We employed a combination of Medical Subject Headings (MeSH) and free-text terms to identify relevant studies. The search strategy incorporated terms related to the disease entities, specific antigens, and therapeutic interventions. The detailed search strings for PubMed provided in Supplementary Table 1.

### 2.2 Inclusion and exclusion criteria

Studies were eligible for inclusion if they met the following criteria: (1) Population: patients diagnosed with peripheral neuropathy based on established clinical and electrophysiological criteria, regardless of age or gender; (2) Serology: confirmation of autoantibodies against nodal/paranodal antigens (NF155, CNTN1, or CASPR1) via validated methods, including Cell-Based Assay (CBA), Tissue-Based Assay (TBA), ELISA, or immunohistochemistry; (3) Intervention: Treatment with at least one cycle or dose of anti-CD20 monoclonal antibody therapy (such as rituximab or ofatumumab).

While the qualitative systematic review included all studies meeting these criteria, the quantitative meta-analysis was restricted to studies providing objective, scale-based outcome data. The primary outcome measure was the proportion of patients achieving a clinical response at the final follow-up. Clinical response was defined as meeting any of the following objective and scale-based outcome data criteria: INCAT reduction ≥1 point, mRS reduction ≥1 point, IRODS increase ≥4 points, MRC increase ≥2 points, ONLS reduction ≥1 point, or NIS improvement.

We excluded patients solely positive for anti-NF186 to maintain phenotypic homogeneity of the study population. Patients with anti-myelin-associated glycoprotein (MAG) antibodies or other identified etiologies were also excluded. Publication types such as reviews, editorials, conference abstracts, and non-English literature were also excluded.

### 2.3 Data extraction

Data extraction was performed using a pre-piloted standardized form. Two independent reviewers (ZJ, T and YH, J) extracted the following variables: demographic details (age, gender, region), clinical characteristics (onset phenotype, disease duration), serological profiles, prior immunotherapy history, anti-CD20 treatment regimens, and clinical outcomes. Any discrepancies were resolved through discussion or adjudication by a third reviewer (QY, G).

### 2.4 Quality assessment

Risk of bias in the included cohort studies was assessed using the Newcastle-Ottawa Scale (NOS) (20). For case reports and case series, the JBI Critical Appraisal Checklist was employed to ensure methodological rigor (21). NOS employs a star rating system with a total score ranging from 0 to 9 stars. Higher scores indicate higher quality. All cohort studies included in the meta-analysis received a 6-star rating (Table 1).

**Table 1:** Quality assessment of included studies in meta-analysis using the Newcastle-Ottawa scale and JBI critical appraisal checklist.

Study	Representativeness of the exposed cohort	Selection of the non- exposed cohort	Ascertainment of exposure	Demonstration that outcome of interest was not present at start of study	Comparability of cohorts on the basis of the design or analysis	Assessment of outcome	Was follow-up long enough for outcomes to occur	Adequacy of follow up of cohorts
Cui et al. (35)	*		*	*		*	*	*
Liu et al. (13)	*		*	*		*	*	*
Hu et al. (18)	*		*	*		*	*	*
Cortese et al. (26)	*		*	*		*	*	*
Rashed et al. (37)	*		*	*		*	*	*
Martín-Aguilar et al. (3)	*		*	*		*	*	*
Delmont et al. (25)	*		*	*		*	*	*

Study	Were patient’s demographic characteristics clearly described?	Was the patient’s history clearly described and presented as a timeline?	Was the current clinical condition of the patient on presentation clearly described?	Were diagnostic tests or assessment methods and the results clearly described?	Was the intervention(s) or treatment procedure(s) clearly described?	Was the post-intervention clinical condition clearly described?	Were adverse events (harms) or unanticipated events identified and described?	Does the case report provide takeaway lessons?
Remiche et al. (43)	Yes	Yes	Yes	Yes	Yes	Yes	Unclear	Yes
Kmezic et al. (40)	Yes	Yes	Yes	Yes	Yes	Yes	Yes	Yes
Talers et al. (45)	Yes	Yes	Yes	Yes	Yes	Yes	Unclear	Yes
Mori et al. (23)	Yes	Yes	Yes	Yes	Yes	Yes	Unclear	Yes
Afanasiev et al. (44)	Yes	Yes	Yes	Yes	Yes	Yes	Unclear	Yes
Bresciani et al. (33)	Yes	Yes	Yes	Yes	Yes	Yes	Unclear	Yes
Wang et al. (31)	Yes	Yes	Yes	Yes	Yes	Yes	Unclear	Yes
Chen et al. (28)	Yes	Yes	Yes	Yes	Yes	Yes	Unclear	Yes
Li et al. (29)	Yes	Yes	Yes	Yes	Yes	Yes	Unclear	Yes
Hu et al. (34)	Yes	Yes	Yes	Yes	Yes	Yes	Unclear	Yes
Wang et al. (30)	Yes	Yes	Yes	Yes	Yes	Yes	Unclear	Yes
Bai et al. (24)	Yes	Yes	Yes	Yes	Yes	Yes	Unclear	Yes
Appeltshauser et al. (32)	Yes	Yes	Yes	Yes	Yes	Yes	Unclear	Yes
Athanasopoulos et al. (39)	Yes	Yes	Yes	Yes	Yes	Yes	Unclear	Yes
Wang et al. (16)	Yes	Yes	Yes	Yes	Yes	Yes	Unclear	Yes
Jamall et al. (22)	Yes	Yes	Yes	Yes	Yes	Yes	Unclear	Yes

Study	Were there clear criteria for inclusion in the case series?	Was the condition measured in a standard, reliable way for all participants included in the case series?	Were valid methods used for identification of the condition for all participants included in the case series?	Did the case series have consecutive inclusion of participants?	Did the case series have complete inclusion of participants?	Was there clear reporting of the demographics of the participants in the study?	Was there clear reporting of clinical information of the participants?	Were the outcomes or follow up results of cases clearly reported?
Lyou et al. (36)	Yes	Yes	Yes	Unclear	Unclear	Yes	Yes	Yes
Hou et al. (17)	Yes	Yes	Yes	Unclear	Unclear	Yes	Yes	Yes
Pascual-Goñi et al. (27)	Yes	Yes	Yes	Unclear	Unclear	Yes	Yes	Yes
Jiao et al. (14)	Yes	Yes	Yes	Unclear	Unclear	Yes	Yes	Yes
Dubey et al. (38)	Yes	Yes	Yes	Yes	Yes	Yes	Yes	Yes
Godil et al. (42)	Yes	Yes	Yes	Unclear	Unclear	Yes	Yes	Yes

In the Newcastle-Ottawa Scale, a study can be awarded a maximum of one star for each numbered item within the Selection and Exposure categories, a maximum of two stars can be given for Comparability. *represents receiving one star.

### 2.5 Statistical analysis

All statistical analyses were conducted using R software (version 4.5.1) with the “metafor” package. Given the anticipated heterogeneity and small sample sizes in rare disease cohorts, we employed a generalized linear mixed model (GLMM) with a logit link function and random intercepts was used to pool single-arm response proportions. Individual studies were treated as random effects to account for between-study variability. This approach allows the inclusion of studies with zero or 100% event rates without the need for continuity correction, thereby reducing potential bias associated with arbitrary adjustments.

Effect sizes were reported as proportions with 95% confidence intervals (CIs). Heterogeneity was quantified using the I^2^ statistic. To evaluate the robustness of our findings, a pre-specified sensitivity analysis was performed by excluding single-case reports. Publication bias was assessed visually via funnel plots and quantitatively using Egger’s regression test for analyses including 10 or more studies. A two-sided *p*-value < 0.05 was considered statistically significant.

## 3 Results

### 3.1 Search results

**Figure 1:**
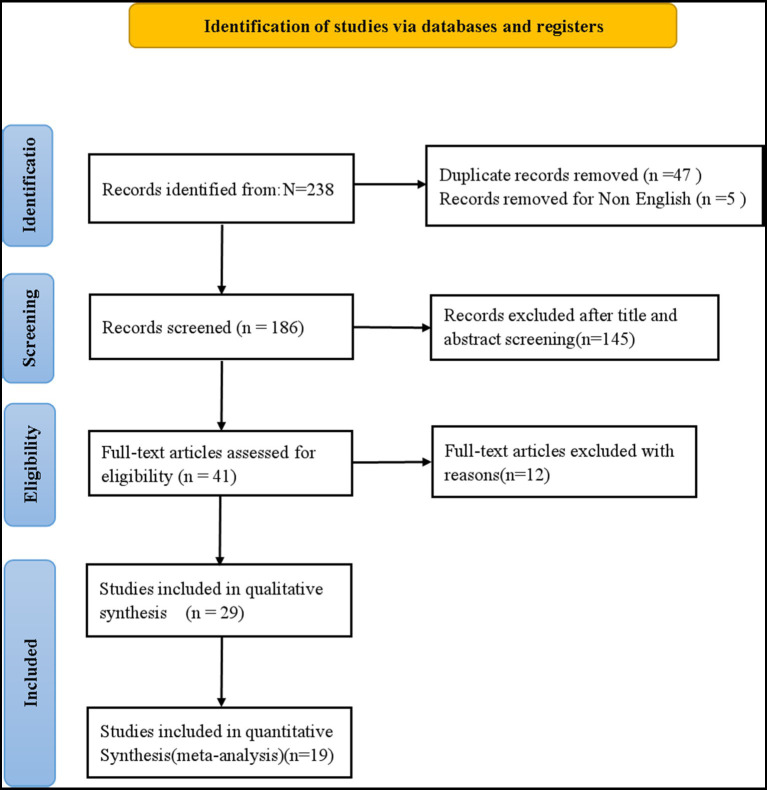
PRISMA flow diagram outlining the study selection process.

Based on the search strategy, 238 records were initially identified. Following the removal of duplicates and screening of titles and abstracts, 41 articles were assessed for full-text eligibility. Twelve studies were subsequently excluded due to non-conforming study designs, irrelevant study populations, or insufficient outcome data. Ultimately, 29 studies (13–17, 22–45) met the inclusion criteria for the systematic review, of which 19 provided sufficient quantitative data for the meta-analysis. The study selection process is detailed in the PRISMA flow diagram (Figure 1).

### 3.2 Characteristics of the included studies

The systematic review comprised 29 studies involving a total of 118 patients. Baseline demographic and clinical characteristics were synthesized from available data (Table 2). The cohort was predominantly male (70/89, 78.7% of evaluable cases) with a mean age of 41.9 years. Clinically, the majority of patients presented with a chronic onset (27/42, 64.3%) and exhibited a typical phenotype (37/53, 69.8%).

**Table 2:** Clinical characteristic of 118 patients in 29 studies.

Study	Study design	Country	No. (male)	Age	CD20 monoclonal therapy type	Dosage regimen	Outcome measures	Co-current diseases
Cui et al. (35)	cohort study	China	5 (3)	10.6 ± 2.07	Rituximab* (plasma exchange)	375 mg/m2 weekly	INCAT, mRS, IRODS	3
Liu et al. (13)	cohort study	China	8 (7)	35.5 (median)	Ofatumumab	20 mg/0.4 mL on day 0, 7, 14, 28, and every 4 weeks	aINCAT, IRODS, MRC	3
Wang et al. (31)	case report	China	1 (0)	70	Ofatumumab* (corticosteroids)	one injection every month	clinical feature	1
Rashed et al. (37)	cohort study	America	10 (unknown)	Unknown	Rituximab	Unknown	NIS, clinical feature	Unknown
Kmezic et al. (40)	case report	Sweden	1 (1)	42	Rituximab	500 mg intravenously (single dose) + a second dose of rituximab (500 mg) was administered 6 months after the prior treatment	INCAT, IRODS, MRC	Unknown
Talers et al. (45)	case report	Latvia	1 (1)	Unknown	Rituximab* (corticosteroids)	1, 000 mg	INCAT, IRODS, MRC, ONLS	Unknown
Mori et al. (23)	case report	Japan	1 (1)	69	Rituximab	375 mg/m2 weekly	INCAT, mRS	1
Chen et al. (28)	case report	China	1 (1)	12	Rituximab	500 mg	clinical feature	Unknown
Appeltshauser et al. (32)	case report	Germany	1 (1)	67	Rituximab* (IVIG, plasma exchange, corticosteroids)	1, 000 mg × 6	clinical feature	1
Liu et al. (13)	cohort study	China	19 (18)	20 (median)	Rituximab	100 mg + 500 mg every 6 months	INCAT, MRC, NIS	Unknown
Afanasiev et al. (44)	case report	Switzerland	1 (0)	53	Rituximab	1 g per course, 2 weeks apart	ONLS	1
Bresciani et al. (33)	case report	Italy	1 (0)	26	Rituximab	375 mg/m2 weekly	clinical feature	Unknown
Li et al. (29)	case report	China	1 (1)	62	Rituximab	Unknown	clinical feature	Unknown
Lyou et al. (36)	case series	Korea	3 (1)	29.3 ± 10.02	Rituximab	375 mg/m2 weekly	mRS	Unknown
Bai et al. (24)	case report	China	1 (1)	29	Rituximab* (5 rounds of plasma exchange)	200 mg × 2 + 500 mg × 3	clinical feature	Unknown
Jamall et al. (22)	case report	America	1 (1)	40	Rituximab* (corticosteroids)	375 mg/m2 weekly	MRC	Unknown
Remiche et al. (43)	case report	Belgium	1 (1)	65	Rituximab	375 mg/m2 weekly	INCAT, IRODS, MRC	1
Martín-Aguilar et al. (3)	cohort study	Multicenter	23 (17)	44.1 ± 20.7	Rituximab	375 mg/m2 every week for 4 consecutive weeks and then monthly for the next 2 months (8), 21 g doses separated by 2 weeks (6), 375 mg/m2 every week for 4 consecutive weeks (6), others (2)	mRS	Unknown
Hu et al. (18)	case report	China	1 (1)	66	Rituximab* (corticosteroids, plasma exchange)	Unknown	clinical feature	Unknown
Wang et al. (30)	case report	China	1 (1)	20	Rituximab* (plasma exchange)	500 mg	MRC, mRS	1
Wang et al. (16)	case report	China	1 (1)	37	Rituximab	375 mg/m2	clinical feature	Unknown
Hou et al. (17)	case series	China	2 (2)	52 ± 2.00	Rituximab	600 mg over two consecutive days, 100 mg on day 1 and 500 mg on day 2	clinical feature	Unknown
Pascual-Goñi et al. (27)	case series	Germany	10 (7)	57.0 ± 10.88	Rituximab	Unknown	mRS	Unknown
Jiao et al. (14)	case series	China	3 (2)	30.7 ± 13.5	Rituximab	100 mg weekly for 4 weeks followed by 100 mg per month for 2 doses	INCAT, mRS, MRC	Unknown
Delmont et al. (25)	cohort study	France	14 (unknown)	Unknown	Rituximab	Unknown	ONLS	Unknown
Athanasopoulos et al. (39)	case report	Germany	1 (1)	27	Rituximab* (IVIG, corticosteroids, plasma exchange)	2 g	INCAT, MRC	1
Dubey et al. (38)	case series	America	2 (unknown)	Unknown	Rituximab	Unknown	INCAT	Unknown
Godil et al. (42)	case series	America	2 (unknown)	Unknown	Rituximab	1,000 mg weekly for 2 weeks, then 1,000 mg every 6 months	clinical feature	Unknown
Cortese et al. (26)	cohort study	Italy	1 (unknown)	Unknown	Rituximab	Unknown	ONLS	Unknown

CBA, Cell-Based Assay; TBA, Tissue-Based Assay; ELISA, Enzyme-Linked Immunosorbent Assay; IVIG, Intravenous immunoglobulin; INCAT, Infammatory neuropathy cause and treatment sensory sumscore; mRS, Modifed Rankin score; MRC, Medical Research Council Sum Score; ONLS, Overall Neuropathy Limitations Scale; IRODS, Inflammatory Rasch-built Overall Disability Scale (IRODS); NIS, Neuropathy Impairment Score; NF155, Neurofascin-155; CNTN1, Contactin-1; CASPR1, Contactin-associated protein 1; *Drug combination.

Serological profiling confirmed antibody status for all 118 patients: 85 (72.0%) were positive for anti-NF155, 18 (15.3%) for anti-CNTN1, and 4 (3.4%) for anti-CASPR1. Notably, rare serological profiles were also identified, including one patient with dual positivity for anti-NF155 and anti-NF186, and 10 patients (8.5%) with antibodies against the CNTN1/CASPR1 complex. Regarding treatment history, IVIG, corticosteroids, or other immunosuppressants were documented in 91 of 118 (77.1%) patients. The predominant anti-CD20 regimen was rituximab (27 studies), typically administered at 375 mg/m^2^ weekly for 4 weeks, while ofatumumab was utilized in two studies.

### 3.3 Effectiveness of anti-CD20 monoclonal antibody treatment

**Figure 2:**
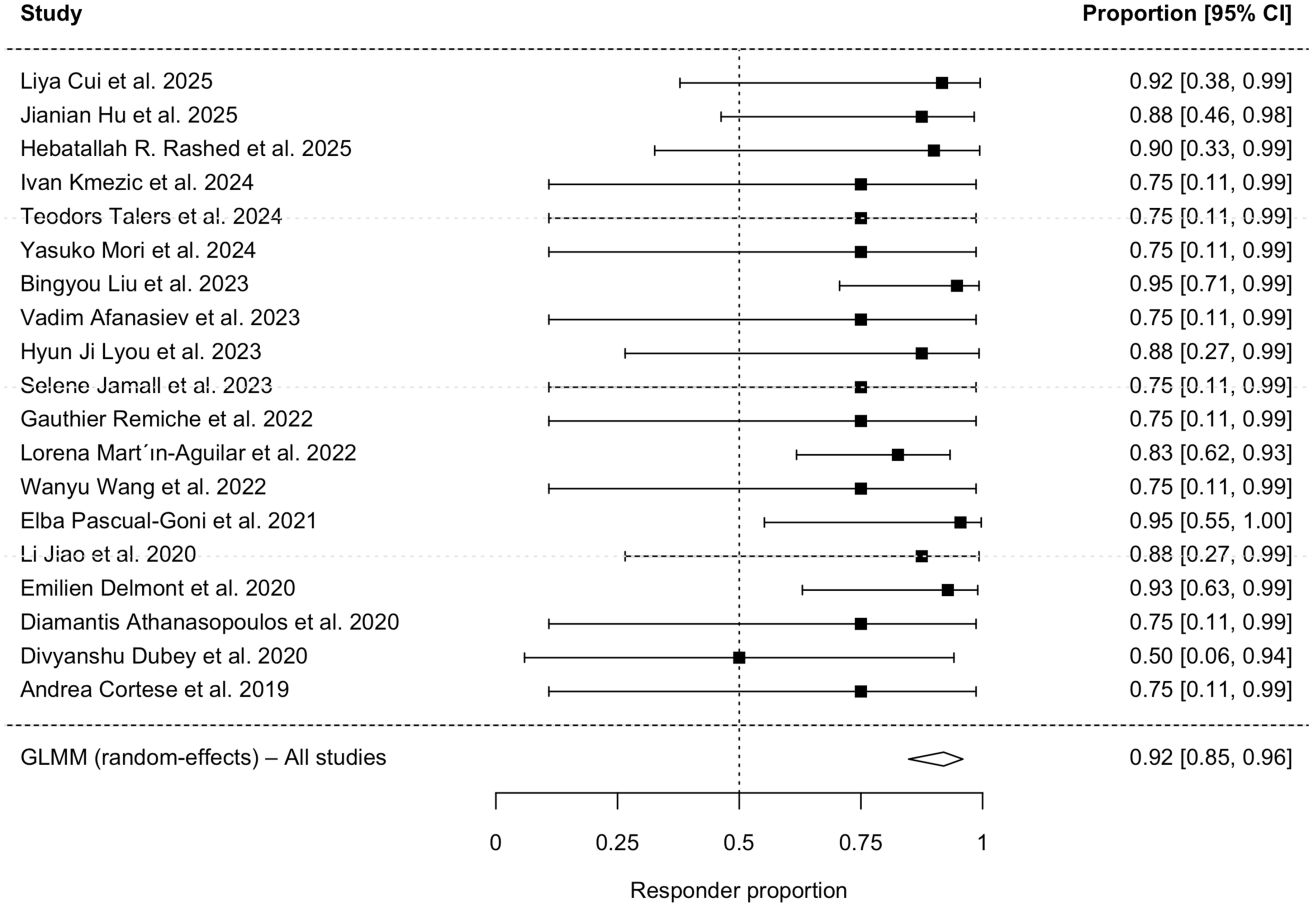
Forest plot of the pooled clinical response rate in patients with AN treated with anti-CD20 monoclonal antibodies. The pooled estimate was calculated using a Generalized Linear Mixed Model (GLMM) with random effects. CI, confidence interval.

**Figure 3:**
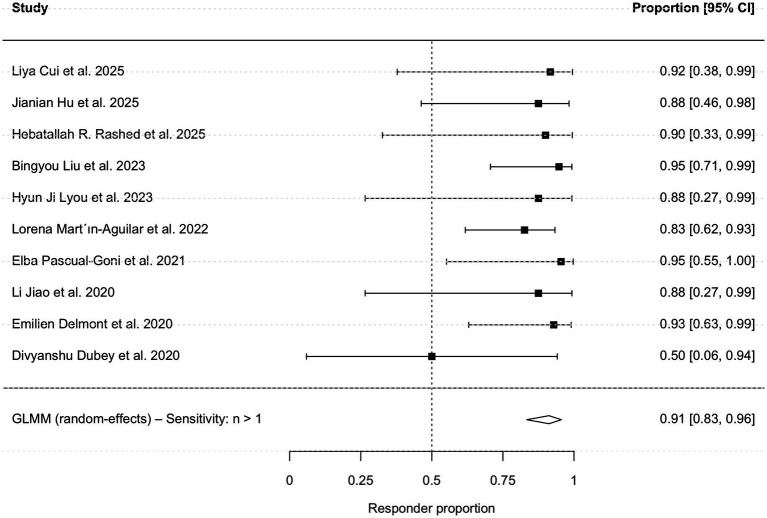
Forest plot of sensitivity analysis of treatment responsiveness. The forest plot displays pooled estimates after excluding single-case reports (retaining 10 studies with n ≥ 2), confirming the robustness of the primary outcome.

First, a descriptive analysis of all 29 studies indicated an overall effectiveness rate of 92.4% based on reported clinical observation improvement. To provide a rigorous quantitative assessment restricted to studies reporting standardized, objective outcome measures, a single-arm meta-analysis was conducted using data from 19 studies (*n =* 100). The clinical characteristic of the patients are shown in Table 3. The pooled clinical response rate was 92.0% (95% CI: 84.8–95.9%; I^2^ = 0%; Figure 2). A sensitivity analysis excluding single-case reports yielded a consistent pooled responsiveness of 91.2% (95% CI: 83.4–95.5%; Figure 3).

**Table 3:** Clinical characteristic of 19 studies included in the meta-analysis.

Study	Study design	No. (male)	Age	Prior treatments	Autoantibody status
Cui et al. (35)	cohort study	5 (3)	10.6 ± 2.07	corticosteroids, IVIG	5 NF155
Hu et al. (18)	cohort study	8 (7)	35.5 (median)	corticosteroids, IVIG, azathioprine, mycophenolate mofetil, rituximab, plasma exchange	7 NF155 and 1 CNTN1
Rashed et al. (37)	cohort study	4 (unknown)	Unknown	IVIG	4 NF155
Kmezic et al. (40)	case report	1 (1)	42	IVIG, corticosteroids	1 NF155
Talers et al. (45)	case report	1 (1)	Unknown	plasma exchange, IVIG, corticosteroids	1 NF155
Mori et al. (23)	case report	1 (1)	69	IVIG, corticosteroids	1 CASPR1
Liu et al. (13)	cohort study	19 (18)	20 (median)	corticosteroids, azathioprine, cyclophosphamide, cyclosporine A, plasma exchange	16 NF155 and 3 CNTN1
Afanasiev et al. (44)	case report	1 (0)	53	IVIG, plasma exchange, corticosteroids, cyclophosphamide, rituximab	1 CASPR1
Lyou et al. (36)	case series	3 (1)	29.3 ± 10.02	IVIG, corticosteroids, azathioprine, plasma exchange	3 NF155
Jamall et al. (22)	case report	1 (1)	40	IVIG, plasma exchange, physiotherapy	1 NF155
Remiche et al. (43)	case report	1 (1)	65	IVIG, methylprednisolone, plasma exchanges	1 CNTN1
Martín-Aguilar et al. (3)	cohort study	23 (17)	44.1 ± 20.7	Unknown	23 NF155
Wang et al. (30)	case report	1 (1)	20	IVIG	1 NF155
Pascual-Goñi et al. (27)	case series	10 (7)	57.0 ± 10.88	IVIG, corticosteroids, plasma exchange, cyclophosphamide	10 CNTN1/CASPR1 complex
Jiao et al. (14)	case series	3 (2)	30.7 ± 13.5	corticosteroids, IVIG, azathioprine, plasma exchange	3 NF155
Delmont et al. (25)	cohort study	14 (unknown)	Unknown	IVIG, corticosteroids, plasma exchange	8 NF155 and 6 CNTN1
Athanasopoulos et al. (39)	case report	1 (1)	27	plasma exchange	1 NF155
Dubey et al. (38)	case series	2 (unknown)	Unknown	Unknown	2 CNTN1
Cortese et al. (26)	cohort study	1 (unknown)	Unknown	IVIG, corticosteroids	1 NF155

IVIG, Intravenous immunoglobulin; INCAT, Inflammatory neuropathy cause and treatment sensory sumscore; mRS, Modified Rankin score; MRC, Medical Research Council Sum Score; ONLS, Overall Neuropathy Limitations Scale; IRODS, Inflammatory Rasch-built Overall Disability Scale (IRODS); NIS, Neuropathy Impairment Score; NF155, Neurofascin-155; CNTN1, Contactin-1; CASPR1, Contactin-associated protein 1.

**Figure 4:**
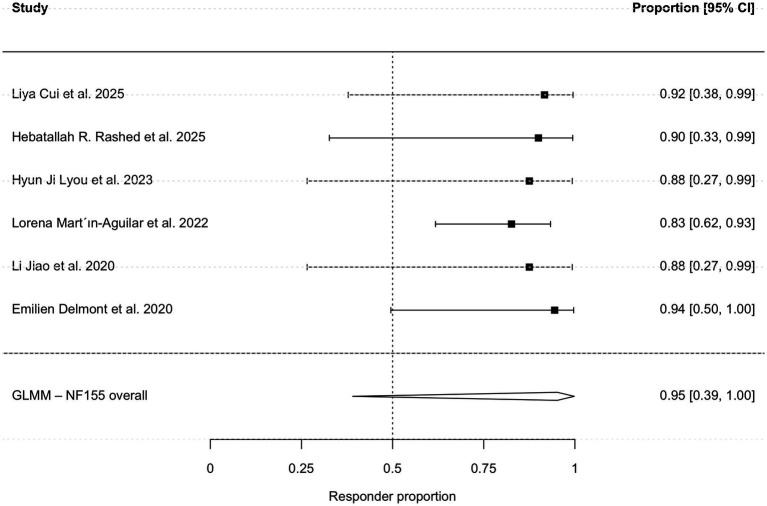
Subgroup analysis of clinical responsiveness in anti-NF155 antibody-positive patients.

**Figure 5:**
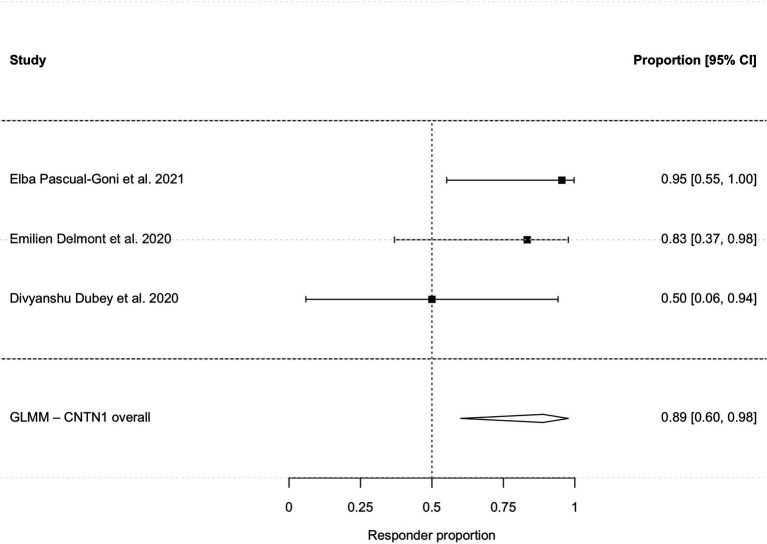
Subgroup analysis of clinical responsiveness in anti-CNTN1 antibody-positive patients.

Subgroup analyses stratified by serostatus revealed high efficacy across key antibody subtypes. The pooled response rate was 95.2% (95% CI, 39.0–99.8%; I^2^ = 34.3%; Figure 4) for the anti-NF155 cohort (*n =* 46) and 88.9% (95% CI, 60.1–97.7%; I^2^ = 16.7%; Figure 5) for the anti-CNTN1 cohort. The clinical characteristic of patients with anti-NF155 antibodies are shown in Table 4.

**Table 4:** Clinical characteristic of patients with anti-NF155 antibodies.

Study	No. (male)	Age	Clinical classification	Physical sign(s), *n* (%)	Intervention period	Evaluation time (months)
Cui et al. (35)	5 (3)	10.6 ± 2.07	Distal type	Ataxia: 5 (100%) Tremor: 1 (20%)	4 weeks	Unknown
Rashed et al. (37)	4 (unknown)	Unknown	Unknown	Unknown	Unknown	Unknown
Lyou et al. (36)	3 (1)	29.33 ± 10.02	Typical type: 1 Distal type: 2	Ataxia: 3 (100%) Tremor: 1 (33.33%)	4 weeks	Unknown
Martín-Aguilar et al. (3)	23 (17)	44.1 ± 20.7	Unknown	Unknown	Unknown	Unknown
Jiao et al. (14)	3 (2)	30.7 ± 13.5	Unknown	Ataxia: 3 (100%) Tremor: 3 (100%)	1 cycle	6
Delmont et al. (25)	8 (unknown)	Unknown	Unknown	Unknown	Unknown	12

NF155, Neurofascin-155.

**Figure 6:**
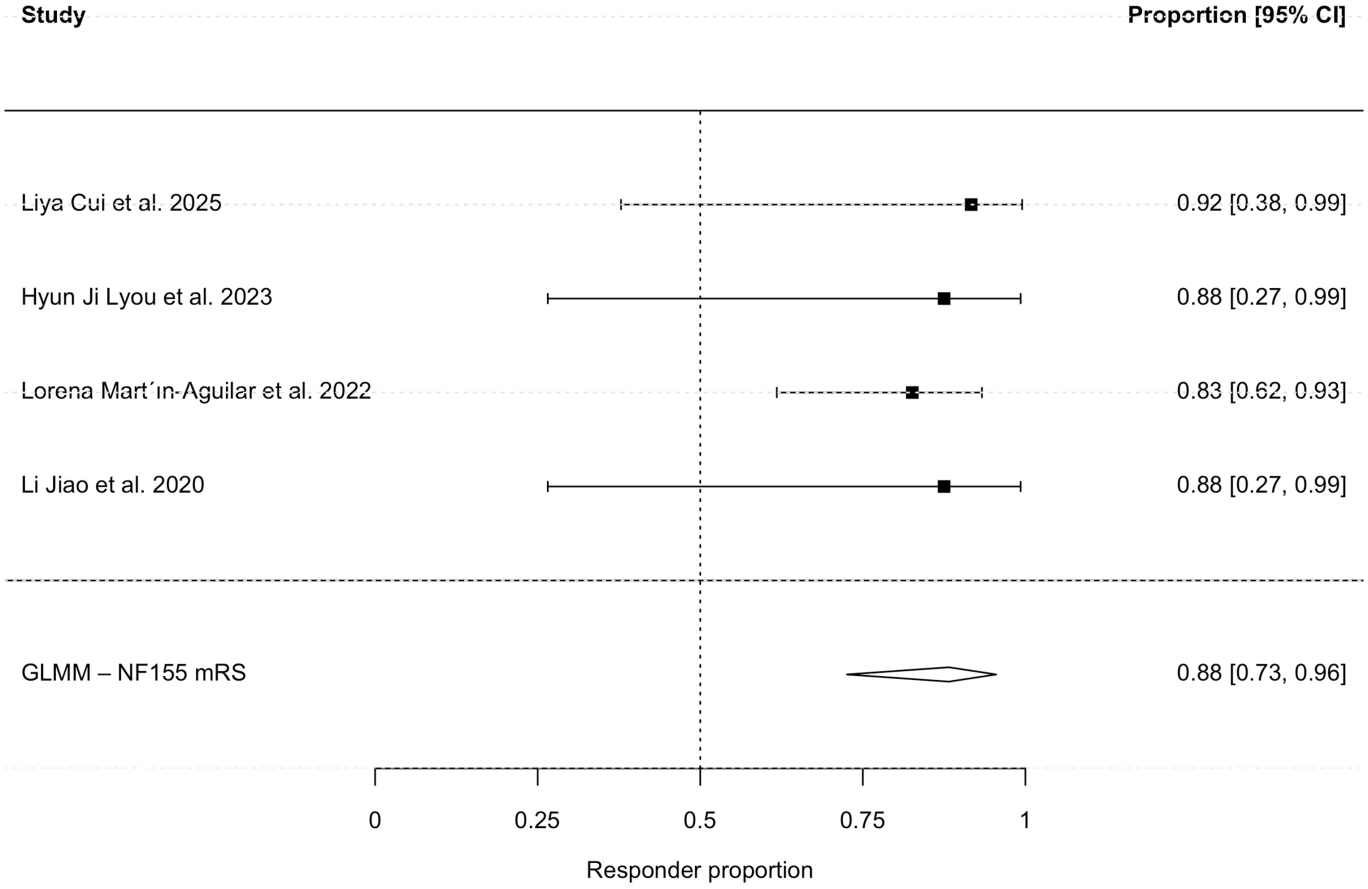
Subgroup analysis based on mRS scale.

To further refine the estimate for anti-NF155 positive patients and mitigate potential bias from subjective scales, a sub-analysis restricted to studies using the mRS demonstrated a robust pooled responsiveness of 88.2% (95% CI, 72.5–95.5%; I^2^ = 0%; Figure 6). Due to the limited sample size, a quantitative meta-analysis was not feasible for the anti-CASPR1 subgroup.

### 3.4 Safety of anti-CD20 monoclonal antibody therapy

Safety data were reported for all 118 patients across the 29 studies. Adverse events (AEs) were documented in 10 patients (8.5%). Most AEs were mild-to-moderate, including infusion-related reactions (IRRs) in five patients (4.2%), upper respiratory tract infections in two (1.7%), and pneumonia in one (0.8%). Notably, two fatalities (1.7%) were recorded, both of which were related to severe infections or comorbidities. The first case involved a patient with refractory autoimmune nodopathy, who developed a disseminated varicella infection following treatment with rituximab (375 mg/m^2^ weekly for 4 consecutive weeks), as well as IVIG and plasma exchange. Despite receiving these treatments, the patient’s infection progressed rapidly, leading to death. The immunosuppressive nature of rituximab, combined with the underlying disease, contributed to the patient’s vulnerability to infections. The second fatality occurred in a bedridden patient with pre-existing membranous glomerulonephritis. This patient, who was treated with rituximab (375 mg/m^2^ weekly for 4 weeks), along with IVIG and corticosteroids, died due to the progression of their kidney disease rather than direct toxicity from the anti-CD20 therapy. Although causality could not be definitively established, these cases underscore the potential risk of serious infectious complications associated with B-cell–depleting therapy.

### 3.5 Publication bias

**Figure 7:**
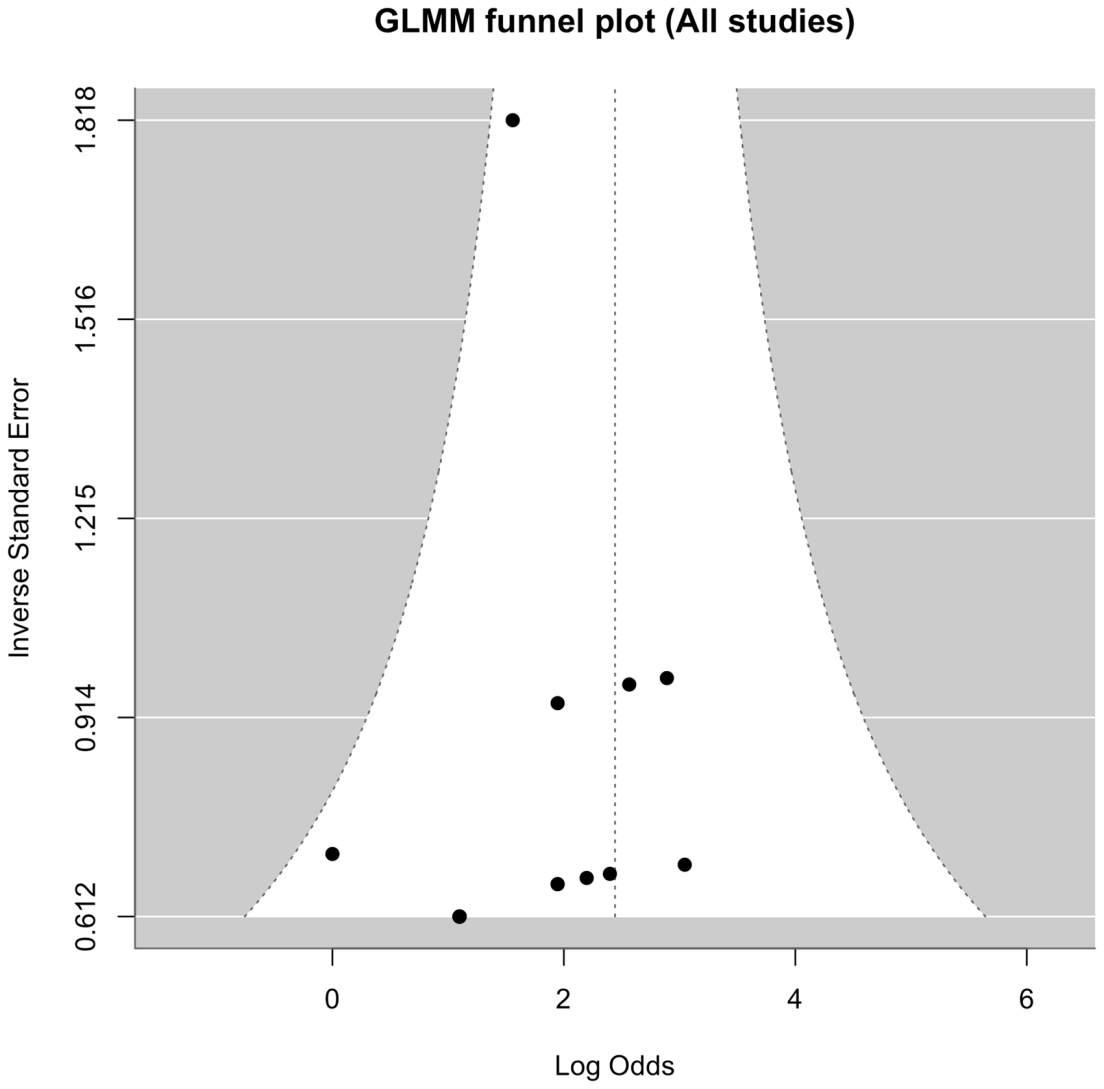
Funnel plot of publication bias. The distribution of the dots indicates the publication bias of the studies.

Evaluation of publication bias was performed for the 10 studies included in the sensitivity analysis. Visual inspection of the funnel plot (Figure 7) revealed a relatively symmetrical distribution. This observation was statistically corroborated by Egger’s regression test, which showed no evidence of significant publication bias (*p* = 0.426).

## 4 Discussion

AN has recently been delineated as a distinct diagnostic entity, characterized by a unique pathophysiology and a marked refractoriness to standard CIDP therapies, such as IVIG. While accumulating case reports and small case series have suggested the efficacy of B-cell depleting agents, existing evidence remains fragmented, and a quantitative synthesis is lacking. Synthesizing data from 19 studies comprising 100 patients, our analysis revealed a pooled clinical response rate of 92.0%. These findings provide the most robust quantitative evidence to date, supporting B-cell depletion as a highly effective therapeutic strategy for AN, particularly for patients harboring anti-NF155 and anti-CNTN1 autoantibodies.

The observed pooled response rate of 92.0% is not only clinically significant but also mechanistically rational. The pathophysiology of AN is increasingly recognized as an IgG4-mediated autoimmune process, distinct from the classical inflammatory demyelination seen in CIDP. Pathogenic IgG4 autoantibodies in AN are believed to be produced primarily by CD20-positive short-lived plasma cells and plasmablasts (46–48). Consequently, anti-CD20 monoclonal antibodies, by depleting this specific B-cell lineage, directly target the upstream source of autoantibody production, thereby abrogating the pathogenic cascade (48, 49). This high efficacy is not an isolated phenomenon; it parallels the established success of B-cell depletion in other IgG4-mediated neurological disorders, such as MuSK-antibody-positive myasthenia gravis and specific forms of autoimmune encephalitis (50–53). This consistency suggests a “positive effect” for anti-CD20 therapies across IgG4-driven auto immunities.

Our demonstrated response rate of 92.0% is notably superior to the 75% reported by Hu et al. (18) in their meta-analysis of general CIDP patients. This discrepancy is likely attributable to differences in patient selection and study population. The meta-analysis by Hu et al. (54) included a heterogeneous cohort of CIDP patients, many of whom were likely seronegative, exhibiting typical macrophage-mediated demyelination that may be less responsive to B-cell depletion. In contrast, our study focused exclusively on serologically defined AN patients. This distinction is further corroborated by the subgroup analysis in Hu et al., which reported that 24 of 25 (96%) anti-IgG4 antibody-positive patients responded to rituximab. Collectively, these findings suggest that anti-CD20 therapy may represent a promising precision medicine approach for AN.

Specifically, the anti-NF155 and anti-CNTN1 subgroups exhibited comparably high response rates (95.2% vs. 88.9%), despite their distinct clinical phenotypes. While anti-NF155 positive patients typically present with tremor and ataxia, anti-CNTN1 positive patients often exhibit aggressive motor involvement and early axonal loss (3, 55). However, their uniform responsiveness to anti-CD20 therapy implies a shared underlying etiology: both conditions are driven by the production of pathogenic IgG4 autoantibodies by B-lineage cells (56, 57). This suggests that B-cell depletion effectively targets the fundamental pathogenesis of the disease across different serological and clinical subtypes.

In our subgroup analysis, the pooled estimate for the anti-NF155 cohort was associated with a wide 95% CI (39.0–99.8%). This statistical instability should not be misinterpreted as therapeutic inconsistency; rather, it is an expected artifact of data sparsity and the limited number of studies available for this specific analysis. To derive a more precise estimate, our pre-specified sub-analysis restricted to anti-NF155 cohorts using the mRS scale yielded a robust response rate of 88.2%. This confirms a high and consistent efficacy, while highlighting the necessity for larger, standardized studies to further refine these estimates.

The safety profile of anti-CD20 therapy in AN appears generally favorable, yet it necessitates strict vigilance regarding infectious risks. Consistent with previous reports, the majority of adverse events observed were mild infusion-related reactions (58–60). However, the occurrence of two fatalities (1.7%) warrants caution. Although opportunistic infections are relatively rare with monotherapy, severe complications such as progressive multifocal leukoencephalopathy (PML) have been reported, particularly in the context of combined immunosuppression (61–64). Consequently, to optimize the risk–benefit ratio, clinicians must carefully manage treatment timing and duration. Rigorous monitoring for infectious complications is mandatory, especially in elderly patients, those with severe comorbidities, or individuals undergoing long-term immunosuppression.

This study has several limitations inherent to meta-analyses of rare diseases. First, all included studies were observational, and the absence of random controlled trials (RCTs) may introduce selection bias. Second, our primary analysis included single-case reports; however, sensitivity analyses excluding these small studies yielded consistent results, supporting the validity of our findings. Furthermore, heterogeneity in treatment regimens and the retrospective collection of safety data may have led to an underestimation of mild adverse events. A key limitation is that IgG subclass determination, particularly for IgG4, was not systematically reported in all studies. While anti-NF155 and anti-CNTN1 antibodies are frequently of the IgG4 subclass, and some evidence suggests CASPR1 antibodies may also be IgG4-mediated, not all case reports provided subclass typing. Therefore, we cannot confirm that all analyzed cases represent IgG4-mediated disease, and our conclusions should be interpreted with caution. Future studies should systematically report antibody subclasses to better define the role of IgG4 in AN.

## 5 Conclusion

In conclusion, anti-CD20 therapy demonstrates high efficacy in AN that are refractory to conventional treatments. However, given the observational nature of the available evidence and the absence of randomized controlled trials, these findings should be interpreted with caution and should not be considered practice-changing at this stage. Prospective controlled studies are required to further define efficacy, optimal dosing strategies, and long-term safety.

## Data Availability

The raw data supporting the conclusions of this article will be made available by the authors, without undue reservation.
