# Supplementary material for: Efficacy and safety of anti-CD20 monoclonal antibody therapy for autoimmune nodopathies: a systematic review and meta-analysis
Source: Front Neurol. 2026 Feb 23;17:1759210. doi: 10.3389/fneur.2026.1759210 (PMC12967985; doi:10.3389/fneur.2026.1759210)
Supplement: Supplementary file 1 [file Table_1.docx]

**Detailed search strings in PubMed**

(("Polyradiculoneuropathy, Chronic Inflammatory Demyelinating" [Mesh] OR "Guillain-Barre Syndrome" [Mesh]) OR ("nodopathy"[Title/Abstract] OR "nodopathies"[Title/Abstract] OR "paranodal"[Title/Abstract] OR "juxtaparanodal"[Title/Abstract]) OR ("Neurofascin"[Title/Abstract] OR "NF155"[Title/Abstract] OR "Contactin 1"[Title/Abstract] OR "CNTN1"[Title/Abstract] OR "Contactin Associated Protein 1"[Title/Abstract] OR "CASPR1"[Title/Abstract])) AND (("Rituximab"[Mesh] OR "Ofatumumab"[Title/Abstract]) OR ("anti-CD20"[Title/Abstract] OR "anti CD20"[Title/Abstract] OR "CD20 antibody"[Title/Abstract] OR "CD20 antibodies"[Title/Abstract] OR "B-cell depletion"[Title/Abstract] OR "B cell depletion"[Title/Abstract]))
